# A Comprehensive Characterization of Simple Sequence Repeats in the Sequenced *Trichoderma* Genomes Provides Valuable Resources for Marker Development

**DOI:** 10.3389/fmicb.2016.00575

**Published:** 2016-04-27

**Authors:** Sahil Mahfooz, Satyendra P. Singh, Ramraje Rakh, Arpita Bhattacharya, Nishtha Mishra, Poonam C. Singh, Puneet S. Chauhan, Chandra S. Nautiyal, Aradhana Mishra

**Affiliations:** ^1^Division of Plant Microbe Interaction, Council of Scientific and Industrial Research-National Botanical Research InstituteLucknow, India; ^2^Maharashtra Institute of Medical Sciences and Research Medical CollegeLatur, India

**Keywords:** *Trichoderma*, comparative genomics, microsatellites, motif conservation, genetic diversity

## Abstract

Members of genus *Trichoderma* are known worldwide for mycoparasitism. To gain a better insight into the organization and evolution of their genomes, we used an *in silico* approach to compare the occurrence, relative abundance and density of SSRs in *Trichoderma atroviride*, *T. harzianum*, *T. reesei*, and *T. virens*. Our analysis revealed that in all the four genome sequences studied, the occurrence, relative abundance, and density of microsatellites varied and was not influenced by genome sizes. The relative abundance and density of SSRs positively correlated with the G + C content of their genomes. The maximum frequency of SSRs was observed in the smallest genome of *T. reesei* whereas it was least in second smallest genome of *T. atroviride.* Among different classes of repeats, the tri-nucleotide repeats were abundant in all the genomes and accounts for ∼38%, whereas hexa-nuceotide repeats were the least (∼10.2%). Further evaluation of the conservation of motifs in the transcript sequences shows a 49.5% conservation among all the motifs. In order to study polymorphism in *Trichoderma* isolates, 12 polymorphic SSR markers were developed. Of the 12 markers, 6 markers are from *T. atroviride* and remaining 6 belong to *T. harzianum*. SSR markers were found to be more polymorphic from *T. atroviride* with an average polymorphism information content value of 0.745 in comparison with *T. harzianum* (0.615). Twelve polymorphic markers obtained in this study clearly demonstrate the utility of newly developed SSR markers in establishing genetic relationships among different isolates of *Trichoderma*.

## Introduction

The genus *Trichoderma* comprises a wide range of species, used as mycoparasitic biocontrol for various agriculturally important crops, including cereals, pulses, vegetables, fruits etc. and the most important species include *Trichoderma atroviride* (*Ta*), *T. harzianum* (*Th*), *T. reesei* (*Tr*), and *T. virens* (*Tv*). *T. atroviride* has demonstrated effective biological control activity against postharvest brown rot of stone fruits, *Rhizoctonia solani* on potato in the field and has provided good protection against *Fusarium culmorum* when applied to wheat seed (Dodd et al., 2003). It is used in the foliar application, seed and soil treatments for suppression of various diseases (Roberti et al., 2000). *T. harzianum* not only grows on plant roots, but its hyphae even penetrates root epidermis, which enhances plant growth and immune responses (Yedidia et al., 1999). *T. virens* has been proven effective against fungal phytopathogens such as *Rhizoctonia solani*, *Pythium ultimum*, *Colletotrichum* spp., *Sclerotinia* spp., among others (Ghisalberti and Sivasithamparam, 1991). In addition, *T. virens* is the active ingredient in the commercial products used in biocontrol applications (Lumsden et al., 1995). *T. reesei* is widely used in industries as a source of cellulases and hemicellulases for the hydrolysis of plant cell wall polysaccharides (Martinez et al., 2008). It is also known to be involved in augmentation of grain amino acids and mineral nutrients by modulating arsenic speciation and accumulation in chickpea (Tripathi et al., 2015).

As importance of these *Trichoderma* species are being realized, their genomes have been sequenced and submitted in public databases (Martinez et al., 2008; Kubicek et al., 2011). To obtain maximum advantage from sequences submitted in public databases, genomics, and bioinformatics tools have grown exponentially. The availability of new tools and applications of existing ones for exploring public databases are opening inexpensive ways to study biological systems. High throughput molecular methods could be developed with the help of these bioinformatics tools and the availability of genome sequences, for the characterization of *Trichoderma* population. Previously, various techniques like RFLP, RAPD and AFLP (Hermosa et al., 2001; Dodd et al., 2004; Buhariwalla et al., 2005; Naef et al., 2006) had been utilized for the genetic characterization of *Trichoderma* isolates, however, most of these techniques have their own limitations due to their reproducibility problems and were found inadequate in assessing within species diversity.

The sequence data generated from the sequencing projects of these fungal species can be mined for the presence of microsatellites or simple sequence repeats (SSRs) in genic (Li et al., 2004; Mahfooz et al., 2012) as well as genomic (Toth et al., 2000; Lim et al., 2004; Kim et al., 2008) portions. These SSRs are useful as a marker for a variety of applications because of their reproducibility, multiallelic nature, codominant inheritance, relative abundance and good genome coverage (Datta et al., 2010). However, despite the many advantages of SSR markers in various biological studies, only few reports (Shahid et al., 2013; Geistlinger et al., 2015) on experimental data on polymorphic SSR markers is still a major limitation for utilizing SSR markers in biological studies in fungal systems especially in mycoparasitic fungi. Apart from their application as molecular markers, SSRs may also help to understand whether these sequences have any functional and evolutionary significance on the basis of its abundance and density in the genome. The genome sequences of different *Trichoderma* species are freely available; however, any formal analysis of SSRs in these sequences is yet to be reported.

Therefore, the present study was undertaken with an objective to study the frequency and distribution of SSRs in whole genome sequences of the four ascomycetes *T. atroviride* (*Ta*), *T. harzianum* (*Th*), *T. reesei* (*Tr*), and *T. virens (Tv*). After retrieving all the SSRs from transcribed sequences, we analyzed sharing of all SSR motifs and also investigated the uniqueness of SSRs within all four species. Furthermore, primers were designed and validated for their potential to estimate genetic diversity within Indian isolates of *Trichoderma*.

## Materials and Methods

### SSR Mining

The whole genome sequences of *T. atroviride*, *T. harzianum*, *T. reesei*, and *T. virens* were downloaded from Department of Energy’s Joint Genome Project^1^. The sequences were obtained in FASTA format. No sequence containing ESTs or cDNA was used in the analysis. The identification of microsatellites was carried out using online software WebSat (Martins et al., 2009). The software searches both perfect and compound SSRs. Repeats greater than 12 bp were considered as SSRs, which means there should be at least six occurrences of a di-nucleotide repeat, four occurrences of a tri-nucleotide repeat, three occurences of a tetra-, penta-, and hexa-nucleotide repeat. All SSRs were analyzed for their frequency of occurrence, density, and relative abundance. Density was calculated by dividing the number of base pairs contributed by each SSR by total length analyzed (Mb). Relative abundance was calculated as the number of SSRs per Mb of a sequence. While scanning di- to hexa-nucleotide SSRs, combinations involving runs of the same nucleotide were considered. In the current analysis, each SSR was considered as unique.

For a better understanding of the evolutionary relationship among the *Trichoderma* species, the sharing of repeats was analyzed within transcribed sequences only. As previously reported in our study (Mahfooz et al., 2015), repeat sharing within the transcripts of *Trichoderma* species was analyzed manually in Microsoft Excel workbook, 2007. All the motifs identified in the transcripts of each species were placed in Microsoft Excel sheet and looked for its counterpart in the transcripts of remaining species. If the motif was present in all the transcript sequences it was deemed as common. Similarly, the motifs shared between two and three transcript sequences were also analyzed. The motif which did not have any match was considered as unique. Primers complementary to the flanking regions of microsatellites were designed using the program PRIMER 3 online software^2^. Primers were 18–24 bp in length, with calculated annealing temperatures of 54–62°C, and expected product lengths of 150–400 bp. A total of 41,556 primers were designed from the four *Trichoderma* species. Primer pairs for 98.7, 84.3, 98.3, and 95.0% of the SSRs from *Ta*, *Th*, *Tr*, and *Tv*, respectively were produced (**Supplementary Tables S1**–**S4**); not all the SSR motifs were located in the regions that are suitable for primer designing. G + C content of the genomes was calculated using online software GC content calculator^3^. Pearson correlation coefficient was calculated using SPSS package (SPSS V16.0, SPSS Inc., Chicago, IL, USA).

### Fungal Isolates

A total of 24 different *Trichoderma* isolates representing 12 each from *T. harzianum (Th)* and *T. viride (Tvi)* were obtained from National Agriculturally Important Microbial Culture Collection (NAIMCC), National Bureau of Agriculturally Important Microorganisms (NBAIM), Mau Nath Bhanjan, Uttar Pradesh, India (**Supplementary Table S5**). These isolates represent different agro-climatic zones of India.

### DNA Isolation and SSR Amplification

A total genomic DNA from 24 *Trichoderma* isolates was extracted using HiPurA^TM^ Fungal DNA Purification Kit (HIMEDIA, India). The PCR was performed in 10.0 μl reaction volume containing PCR buffer (10 mM Tris-HCl pH 9.0, 1.5 mM MgCl_2_, 50 mM KCl, 0.01% gelatine), 200 μM each of dNTP (Merck), 0.2 U of Taq DNA polymerase (Merck), 10 pM each of forward and reverse primers, and 10 ng of genomic DNA as template was used in PCR tubes. PCR program was as follows: after initial denaturation at 95°C for 3 min, five touch-down PCR cycles comprising of 94°C for 20 s, 60/55°C for 20 s, and 72°C for 30 s were performed. These cycles were subsequently followed by 40 cycles of denaturation at 94°C for 20 s with a constant annealing temperature of 54–60°C (depending on primer) for 20 s, and extension at 72°C for 20 s, and a final extension at 72°C for 20 min. All PCR amplicons were resolved by electrophoresis on 2% agarose gel to identify the informative SSR loci across all the isolates.

### Statistical Analysis

The amplification data generated by SSR primers was analyzed using SIMQUAL (Nei and Li, 1979) to generate a Jaccard’s similarity coefficient (Jaccard, 1908) using NTSYS-PC, software version 2.1 (Rohlf, 1998). These similarity coefficients were used to construct a dendrogram depicting genetic relationships among the isolates by employing the Unweighted Paired Group Method of Arithmetic Averages (UPGMA) algorithm and SAHN clustering. The allelic diversity or polymorphism information content (PIC) was measured as described earlier (Botstein et al., 1980). PIC is defined as the probability that two randomly chosen copies of a gene will represent different alleles within a population. The PIC value was calculated with the formula as follows:

$$PIC_{i}=1-\overset{n}{\underset{j=1}{\Sigma }}Pij$$

where P_ij_ represents the frequency of the j^th^ pattern for marker i, and summation extends over n patterns.

## Result and Discussion

### Relative Abundance and Relative Density of SSRs

**FIGURE 1:**
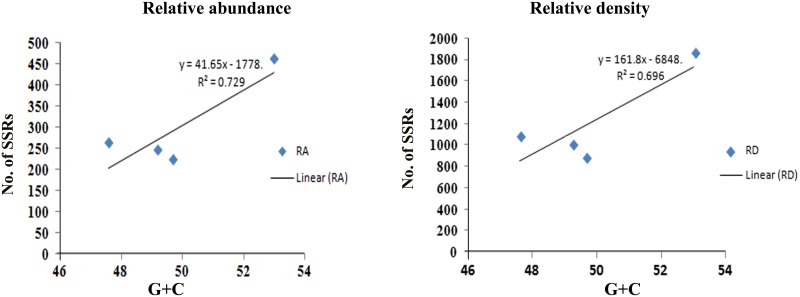
**Figure showing positive correlation of G + C content with relative abundance and density of SSRs**.

The maximum frequency of SSRs among the sequence sets were identified in the genome sequence of *Tr* (15,768) followed by *Th* (10,746), *Tv* (9,535), and *Ta* (8,038). We observed that *Th* which had largest genome size showed second highest frequency of SSRs, whereas, despite having least genome size, the frequency of SSRs in *Tr* was maximum. Therefore, to compare the total SSR count among all four genomes more realistically, we have taken 1 Mb length of each set of sequences analyzed as a reference. Therefore, total relative abundance and total relative density were calculated. It was found that relative abundance and density of SSRs in *Tr* (462.4 and 1868.1) was much higher in comparison to *Ta* (222.6 and 888.4), *Tv* (245.7 and 1036.4), and *Th* (262.7 and 1093.0). It is noteworthy that the total length covered by the SSRs in the genome of *Tr* was twofolds higher when compared with the other species (**Table 1**). To find an appropriate justification for higher relative abundance and density in *Tr*, we looked into the genomic organization of *Trichoderma* species. Genome nucleotide content is one of the key factors which are responsible for the generation of SSRs (Tian et al., 2011). It has been reported that many SSRs first arise by chance substitutions that make a short repetitive sequence, which can undergo slippage if they are above a threshold (Bachtrog et al., 1999). Generation of these initial short random SSRs is easier with biased nucleotide compositions which means that genomes with high GC or AT content have higher SSR densities as compared to genomes having balanced AT/GC content (Tian et al., 2011). The higher relative abundance and density of SSRs in *Tr* prompted us to look at the G + C content of the genomes. Surprisingly, the G + C ratio of *Tr* (53%) was much higher when compared with *Th*, *Tv*, and *Ta* (∼48%). A positive correlation between G + C content of the genome with relative abundance and density of SSRs was also observed (**Figure 1**). Although, G + C content explains a large fraction of variation in relative abundance and density within species, other factors like difference in slippage rate mutations (Viguera et al., 2001) might also explain some of the variations among different species. It is also possible that higher SSR count in *Tr* may belong to the regions without synteny to other genomes which contain genes that are important for the adaptation (Kubicek et al., 2011).

**Table 1:** Occurrence, relative abundance and relative density of SSRs in whole genome sequences of *Trichoderma* species.

	*T. atroviride*	*T. harzianum*	*T. reesei*	*T. virens*
Genome size (Mb)	36.1	40.98	34.1	38.8
No. of chromosomes	6	6	6	6
G + C (%)	49.7	47.6	53.0	49.2
Number of SSR identified	8038	10746	15768	9535
Perfect	7530 (93.68%)	9961 (92.69%)	13868 (87.95%)	8742 (91.68%)
Compound	497 (6.18%)	773 (7.19%)	1847 (11.71%)	772 (8.09%)
Total length of SSR (bp)	32073	44705	63703	40216
% length in genome	0.08	0.10	0.18	0.10
Relative abundance	222.6	262.7	462.4	245.7
Relative density	888.4	1093.0	1868.1	1036.4


We further analyzed the data for the occurrence of different classes of motifs. Tri-nucleotide motifs were undoubtedly the most common motifs in the genomes of *Trichoderma* species and account for almost 38%, however, its percentage was significantly higher in *Tr* when compared with remaining genomes (**Table 2**). Tetra-nucleotide motifs were the second most abundant motifs in *Trichoderma* species whereas hexa-nucleotide motifs were the lowest. The occurrence of higher tri-nucleotide motifs in the sequence can be explained on the basis of their localization. A recent report indicated a clear dominance of tri-nucleotide repeats and a selection against other repeat number, i.e., di- and tetra-nucleotides, both in regions inside open reading frames and in upstream 5′ untranslated regions. Moreover, motifs were more conserved in these regions within species as compared to SSR in other regions, suggesting their evolution is constrained by the functions of the regions they are in (Gonthier et al., 2015). Furthermore, suppression of non-trimeric SSR is also reported in the coding regions due to frame shift mutation (Metzgar et al., 2000). Previous studies have also reported the higher abundance of tri-nucleotide repeats in the transcript sequences of fungi, (Garnica et al., 2006; Mahfooz et al., 2015) plants, (Lawson and Zhang, 2006), and humans (Subramanian et al., 2003).

**Table 2:** Percentage, relative abundance, and relative density of SSRs in whole genome sequence sets of different species of *Trichoderma.*

	Class	Count	Percentage	RA	RD
*T. atroviride*	Di	923	10.7	25.5	51.1
	Tri	3473	40.3	96.2	288.6
	Tetra	2200	25.5	60.9	243.7
	Penta	1064	12.3	29.4	147.3
	Hexa	948	11.0	26.2	157.5

*T. hazarium*	Di	945	8.1	23.0	46.2
	Tri	4016	34.4	97.9	294.5
	Tetra	3905	33.4	95.2	381.9
	Penta	1617	13.8	39.4	197.6
	Hexa	1177	10.0	28.7	172.6

*T. reesei*	Di	2408	13.3	70.6	141.2
	Tri	8262	45.7	242.2	726.8
	Tetra	4333	23.9	127.0	508.2
	Penta	1573	8.7	46.1	230.6
	Hexa	1484	8.2	43.5	261.1

*T. virens*	Di	1074	10.2	27.6	55.3
	Tri	3550	33.9	91.4	274.4
	Tetra	2960	28.3	76.2	305.1
	Penta	1642	15.7	42.3	211.5
	Hexa	1228	11.7	31.6	189.8


Among the tri-nucleotide repeat, motifs AAG/CTT were the most common motifs in *Ta* and *Tv* (3.7 and 3.09%) whereas in *Th* it was TAA/TTA which was most favored (4.33%). Surprisingly, *Tr* showed preference for di-nucleotide motifs AG/CT (3.79%) (**Table 3**). Higher abundance of CAG and AAG repeats is expected in *Trichoderma* species. In our earlier study, similar preference of these repeats in transcripts of different *Fusarium* species were also observed (Mahfooz et al., 2015). Our result indicates that higher abundance of these motifs is the characteristic feature of the genus; however, further investigations on other members of these genera are requisite to come to any strong conclusion. The higher abundance of AG/CT di-nucleotide motif in *Tr* may be explained on the basis that these motifs are less prone to mutation compared to CA/TG or other higher mutable di-nucleotide repeats (Guo et al., 2009). These repeats may be selectively eliminated to reduce mutational pressure exerted on these di-nucleotide repeats. The longest repeat units observed were of di-nucleotides, AG repeated 45 times in *Tr* while AC repeated 40 times in *Tv* (**Table 4**). Overall, our results indicated preference for shorter repeats in the genomes of *Trichoderma* species. This may be explained by the fact that SSRs with a greater repeat number may be more unstable due to the increased probability of slippage (Ellegren, 2004).

**Table 3:** Most common repeat motif identified from perfect and compound microsatellite in the whole genome sequence of four *Trichoderma* species.

*T. atroviride*	Count	*%*	*T. harzianum*	Count	*%*	*T. reesei*	Count	*%*	*T. virens*	Count	*%*
aag/ctt	315	3.7	taa/tta	505	4.33	ag/ct	686	3.79	aag/ctt	324	3.09
gaa/ttc	312	3.6	gaa/ttc	307	2.63	ga/tc	656	3.63	gaa/ttc	316	3.02
aga/tct	276	3.2	aag/ctt	285	2.44	cag/ctg	568	3.14	ag/ct	298	2.8
cag/ctg	258	3	ata/tat	259	2.2	gca/tgc	505	2.79	ga/tc	282	2.69
ga/tc	253	2.9	aga/tct	250	2.14	agc/gct	489	2.70	aga/tct	257	2.45
ag/ct	247	2.86	taaa/ttta	249	2.13	gaa/ttc	448	2.4	cag/ctg	212	2.02
agc/gct	243	2.82	ag/ct	230	1.97	aag/ctt	445	2.46	gca/tgc	211	2.01
gca/tgc	242	2.81	aata/tatt	216	1.85	ctc/gag	438	2.4	agc/gct	203	1.94
ctc/gag	141	1.63	ataa/ttat	216	1.85	gga/tcc	423	2.34	ctc/gag	193	1.84
ta	138	1.6	ga/tc	214	1.83	aga/tct	417	2.3	at	148	1.41
cga/tcg	119	1.38	cag/ctg	204	1.74	ca/tg	414	2.29	gga/tcc	140	1.33
gcc/ggc	119	1.38	atta/taat	194	1.66	ac/gt	342	1.89	taa/tta	138	1.32
cgc/gcg	111	1.28	gca/tgc	184	1.58	agg/cct	333	1.84	ata/tat	125	1.19
gaaa/tttc	108	1.25	aaat/attt	182	1.56	taa/tta	301	1.66	ta	123	1.17
gga/ttc	108	1.25	ctc/gag	182	1.56	gac/gtc	276	1.52	cga/tcg	117	1.11
gac/gtc	103	1.19	ttaa	179	1.53	ctac/gtag	274	1.51	tca/tga	114	1.09
ca/tg	101	1.17	aat/att	171	1.46	gcc/ggc	268	1.48	aaat/attt	111	1.06
at	98	1.13	ta	154	1.32	caa/ttg	264	1.46	agg/cct	111	1.06
aaag/cttt	93	1.08	agc/gct	139	1.2	tca/tga	260	1.43	ac/gt	108	1.03
ccg/cgg	93	1.08	gga/tcc	137	1.17	cga/tcg	258	1.42	ca/tg	107	1.02
tca/tga	88	1.02	ca/tg	129	1.10	ggta/tacc	255	1.4	caa/ttg	106	1.01
caa/ttg	87	1.01	tca/tga	115	0.98	ccta/tag	243	1.34	gaaa/tttc	106	1.01
atg/cat	83	0.96	gaaa/tttc	114	0.97	atg/cat	234	1.29	aaag/cttt	105	1.00
cca/tgg	83	0.96	at	113	0.96	gta/tac	231	1.27	taaa/ttta	105	1.00
ac/gt	78	0.91	gcc/ggc	106	0.90	cgc/gcg	223	1.23	gcc/ggc	100	0.95
aaga/tctt	71	0.82	agg/cct	104	0.89	ata/tat	196	1.08	aat/att	92	0.88
cac/gtg	66	0.76	cga/tcg	102	0.87	aca/tgt	188	1.04	atg/cat	89	0.85
atc/gat	63	0.73	atc/gat	99	0.84	ccg/cgg	186	1.02	aaga/tctt	84	0.80
agaa/ttct	62	0.72	atg/cat	99	0.84	cta/tag	179	0.99	ataa/ttat	83	0.79
cta/tag	62	0.72	aaag/cttt	95	0.81	cca/tgg	177	0.98	cag/gtc	83	0.79


**Table 4:** The longest SSR motifs found in the whole genome sequences of four *Trichoderma* species analyzed.

Longest motifs	*T. atroviride*	*T. harzianum*	*T. reesei*	*T. virens*
Di-nucleotide	(tc)_38_,	(tc)_28_, (ga)_28_	(ag)_45_	(ac)_40_
Tri-nucleotide	(cta)_27_,(ctt)_27_	(tat)_31_, (att)_31_, (caa)_31_	(gga)_38_, (aag)_38_	(ctc)_36_
Tetra-nucleotide	(gctc)_11_	(tatt)_23_, (aaat)_23_	(ttat)_34_	(ttct)_30_
Penta-nucleotide	(tgaag)_11_	(aaaat)_13_	(agcag)_10_	(tttct)_27_
Hexa-nucleotide	(ttgctt)_19_	(ttattt)_13_	(ggttag)_13_	(tggaag)_19_


### Conservation of SSR Motifs within Species

**FIGURE 2:**
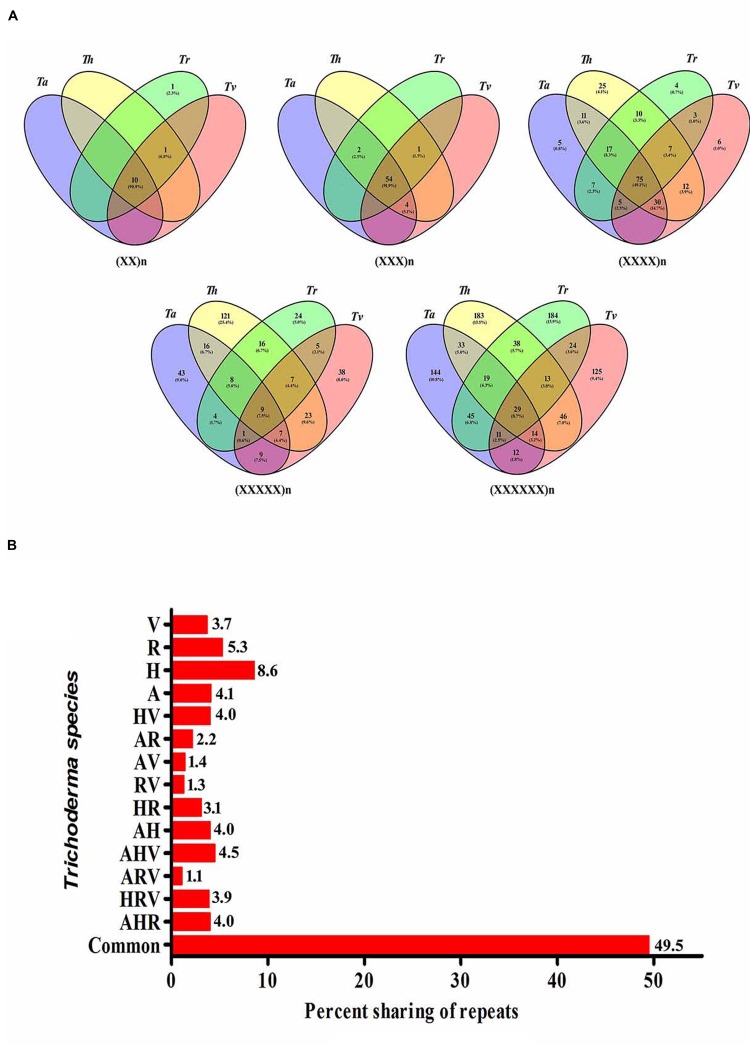
**(A)** Vinn diagram showing sharing of different classes of motifs in the transcripts of *Trichoderma* species. **(B)** Graphical representation of sharing of all types of motifs in the transcripts of *Trichoderma* species (A, *T. atroviride*; H, *T. harzianum*; R, *T. reesei*; V, *T. virens*).

To study the evolutionary relationships among the four *Trichoderma* species and to identify unique motifs, we further scanned the transcripts of the *Trichoderma* species for the occurrence of SSRs. The maximum relative abundance and density of SSRs were observed in *Tr* followed by *Th*, *Ta*, and *Tv*. Each motif was analyzed for the presence of its counterpart in the remaining species. Maximum number of motifs shared between all four transcripts was tri-nucleotide repeats (54, 91.1%) followed by di-(10, 90.9%), tetra-(75, 49.1%), hexa-(29, 8.7%), and penta-nucleotide repeats (9, 7.5%) (**Figure 2A**). While comparing the sharing of all classes of repeat motifs between two transcripts, we observed that *Th–Tv* and *Ta–Th* shared highest percentage of motifs (4.0%) whereas least sharing (1.3%) was observed between *Tr* and *Tv* (**Figure 2B**). Among three transcripts, it was the mycoparasitic trio of *Ta–Th–Tv* that shared a maximum number of motifs. The sharing of the maximum number of motifs between *Th–Tv*, *Ta–Th*, and *Ta–Th–Tv* is therefore not very surprising. *Ta–Tv* shares 1273 orthologs that are not present in *Tr* (Martinez et al., 2008) and this may be contributing to the mycoparasitic activity in these species. Our result indicates that the distribution of SSR motifs does not follow a genus wide pattern, it is strictly species dependent and this could reflect phylogenetic relationship among the species. Unique motifs were also observed among individual transcripts within all classes of motifs excluding tri-nucleotide motifs (**Supplementary Table S6**). It is important to note that *Th* harbors a maximum number of tetra- and penta-nucleotide motifs whereas *Tr* had the highest number of hexa-nucleotide motifs. Maximum occurrence of unique motifs was observed in the transcripts of *Th* (8.6%) which was followed by *Tr* (5.3%) and *Ta* (4.1%). The least number of unique motifs were found in *Tv* (3.7%) (**Supplementary Table S6**). Among all, 49.5% motifs were conserved within all four transcripts (**Figure 2B**). Our previous analysis in *Fusarium* resulted in 36.5% conservation of motifs (Mahfooz et al., 2015) which indicated a lesser genomic heterogeneity in the mycoparasitic *Trichoderma* compared to the pathogenic *Fusarium*.

### Codon Usage

The tri-nucleotide motifs which are most abundant in the transcripts of *Trichoderma* species were further analyzed for amino acids encoded by them. In our analysis, we observed that motifs coding for arginine were dominating in all the species (**Supplementary Table S7**). The second most abundant motif codes for alanine and glutamine, alanine in *Ta* and *Tv* and glutamine in *Th* and *Tr*. The higher abundance of arginine is expected because arginine has a reputation of being among the major nitrogen sources in fungi (Kaur et al., 2011). Apart from that, arginine is also reported for conidiation in some fungi (Gong et al., 2007). The wide presence of glutamic acid and glutamine is justified by the fact that they play an important role in nitrogen regulatory pathway as a metabolic repressor and mainly used as a nitrogen source by fungi. Nitrogen metabolic regulation appears to have a significant role in the pathogenicity of certain animal and plant fungal pathogens (Marzluf, 1997). We also computed the Pearson correlation coefficients among different amino acid repeats within the species. Maximum correlation (*r* = 0.989) was observed between *Ta* and *Th* whereas *Tr* and *Tv* correlated the least (*r* = 0.921). The value indicates that different amino acids encoded by tri-nucleotide SSRs tend to occur in the same genome. The composition biased between *Tv* and *Tr* may be explained on the basis of hybridization of two related species with distinct GC content (Payen et al., 2009). Here, it is interesting to note that the correlation of amino acid repeats matches well with the percentage sharing of repeats within two species.

### Evaluation of Genic-SSR Markers for Polymorphism Analysis

A total 20 genic-SSR primers representing 10 each from *Ta* and *Th* were randomly selected and used for PCR amplification to study their utility in genetic diversity analysis of *Trichoderma* isolates obtained from different geographical locations in India. Of the total primers, only 12 primers amplified fragments in all the 24 isolates of *Trichoderma.* The remaining eight primers did not amplify even with changes in PCR conditions; therefore, these primers were not included for polymorphism studies. The remaining 12 primers amplified easily scorable bands of 130–410 base pair size range (**Table 5**). Among the markers, four amplified di-nucleotide repeats, seven amplified tri-nucleotide repeats, and one marker amplified tetra-nucleotide repeat. A total of 34 alleles were amplified by the 12 markers with an average of 2.8 alleles per locus. *Ta* markers amplified 12 alleles with an average of 2 alleles per locus, whereas markers from *Th* amplified 22 alleles with an average of 3.6 alleles per locus. A recent study in *Tv* has detected 7.4 alleles per locus by using SSR markers (Geistlinger et al., 2015). The higher number of alleles obtained in their study might be attributed to the fact that the authors have used global collection of *Tv* whereas we have restricted only to the Indian isolates. Among the polymorphic markers, maximum PIC value was obtained with primer *Ta*1394 (0.97), whereas minimum PIC value was obtained with primer *Th*79 (0.15), the average being 0.68. Markers with high PIC value (*Ta*1394 and *Ta*1195) will be highly informative for genetic diversity studies and are extremely useful in distinguishing the polymorphism rate of the marker at the specific locus.

**Table 5:** Detail of locus, primer sequence, Tm, Motif, Alleles size, percentage polymorphism, No. of alleles, and PIC value of different primers used to evaluate genetic diversity within *Trichoderma* isolates.

	Forward (5′-3′)	Reverse (5′-3′)	Tm °C	Motif	Allele size	% Poly-morphism	No. of alleles	PIC
Ta 1195	attgacgactcatagcgttt	ttttcagtcaggtctcgttt	54.9	(agac)9	300–320	100	2	0.95
Ta 1394	gccttacttgaagccactaa	ctagcttgcctgcttatgat	55.0	(ga)8-(at)6	200–500	100	2	0.97
Ta 1644	gacgtctcgaatcttcaatc	atgtgacaattgaacagcaa	54.8	(tga)_11_	250	100	1	0.37
Ta 2172	atgtacagtagcaccgctct	ttgtttgctttctggacttt	55.1	(gca)_15_	350–600	100	4	0.67
Ta 2383	accaccagcgcattct	gttggttcgtctgagatagc	55.5	(ca)_20_	350	100	1	0.71
Ta 2584	agctcgatgtctatagcagg	tattgagctcatccttggtt	54.8	(ctg)_9_	350–400	100	2	0.79
Th79	gtacagcagcaattcgtaca	cactgatggtgcatattgag	54.9	(aca)_21_	150–500	100	5	0.15
Th109	tgcatctctccagattttct	tccagaactccatccataac	54.9	(ct)_9_	200–400	100	2	0.96
Th276	ctaagcctacggacaagaag	gttgccgttgttgttattg	55.0	(aac)_29_	375–600	100	4	0.44
Th697	gctggcattaggtacagaac	ttagtcaaggctttttgcat	55.1	(aca)_12_	275–300	100	2	0.71
Th1583	caggtactattcgtaggcgt	ttctgaggttgaaggtgact	54.6	(tct)_14_	200–500	100	4	0.75
Th2067	tggcaatgtctctctctttt	ttttgcttctcttcctcttg	55.0	(tc)_16_	120–900	100	5	0.69


**FIGURE 3:**
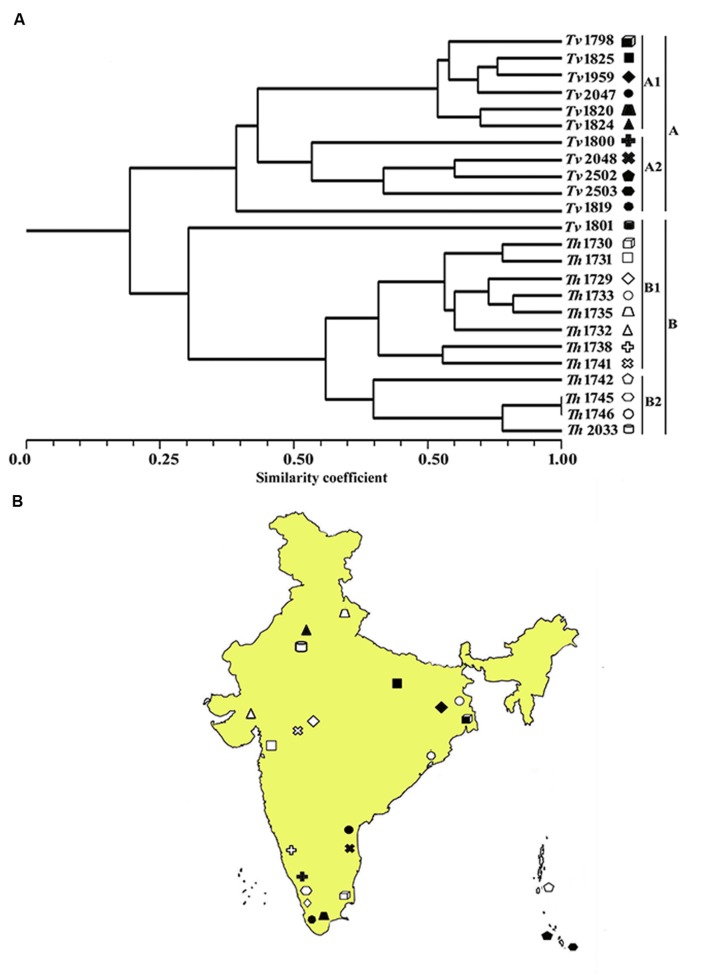
**(A)** Dendrogram showing genetic relationship among the *Trichoderma* isolates based on 12 microsatellite markers. Scale indicates Jaccard’s coefficient of similarity. A and B indicates main clusters. 1A, 2A, 1B, and 2B indicate sub-clusters within main cluster A and B. **(B)** Map of India showing the geographical location of different isolates used for diversity analysis in this study.

In order to quantify the level of polymorphism, Jaccard’s estimate of similarity based on the probability that an amplified fragment from one isolate will also be found in another was used to generate a similarity matrix. The similarity coefficient values between isolates ranged from 0.1 to 0.91 with a mean of 0.56. For markers derived from *Th*, the similarity coefficient values between isolates were in the range from 0.12 to 0.90 with an average genetic diversity of 48%. Similarly, for markers from *Ta* the similarity coefficient values were in range from 0.18 to 0.89 with an average genetic diversity of 42% (**Table 6**). In an earlier study, based on rDNA and PCR-RAPD profiles, molecular characterization of *Tvi* and *Th* isolates from Bengal region of India revealed a similarity coefficient ranging from 0.67 to 0.95 (Chakraborty et al., 2010). The wide range of similarity coefficient values obtained in our study might be attributed to the unique mechanism responsible for generating microsatellite allelic diversity by replication slippage rather than by simple mutation (Viguera et al., 2001). This suggests that the isolates used in this study are genetically more diverse as they were collected from different geographical regions of India. Based on the values of similarity index, a dendrogram was constructed which resulted into two main clusters A and B. Both the clusters were further subdivided into two sub-clusters. Sub-cluster A1 and A2 exclusively comprised of *T. viride* (*Tvi)* isolates whereas cluster 2A and 2B grouped all the isolates of *Th* with an exception of isolate *Tv*1801 which also showed similarity with *Th* isolates and grouped along with them forming a distinct cluster (**Figure 3A**). The grouping of *Tvi* isolate 1801 along with *Th* isolates was surprising. However, when we looked into the geographical localization of these isolates, we observed that theses isolates came from south Kerala region of India where geographical and climatic conditions remain same almost throughout the year (**Figure 3B**). This might have resulted in a narrow genetic variability within the isolates which is reflected in our study (Olango et al., 2015).

**Table 6:** A comparison between *T. atroviride* and *T. harzianum* markers in order to estimate the level of polymorphism revealed by them.

	*Ta*	*Th*	All
Markers used	10	10	20
Marker amplified	6	6	12
No of polymorphic markers	6	6	12
No of monomorphic markers	–	–	
Average PIC value	0.745	0.615	0.680
No. of alleles amplified	12	22	34
Similarity coefficient value (Avg)	0.57	0.52	0.563


## Conclusion

The population structure can vary through origin and space as the organism evolve or adapt in response to environmental conditions. Hence, molecular characterization of an organism is required. To this effect we have tried to gain some insight into the molecular characterization of *Trichoderma* species through SSRs. We found evidence that the frequency of SSRs in genus *Trichoderma* is correlated with the GC content of their genomes. With the identification of numerous unique motifs, the current findings should stimulate the efforts to develop species specific microsatellite markers in *Trichoderma*. Furthermore, on the basis of sharing of repeats, we observed higher conservation of motifs in *Trichoderma* which hinted a homogeneous genome organization as compared to other ascomycetes. Overall, this work on marker development and diversity analysis of *Trichoderma* species will provide a better identification of *Trichoderma* strains with biocontrol mechanism for the development of suitable bio formulation in sustainable agriculture.

## Author Contributions

SM, SS, and RR generated and analyzed the bioinformatics data. AB, NM performed the wet lab experiments. SM, PS, and PC contributed to the writing of the manuscript. AM and CN directed and oriented the project and revised the manuscript.

## Conflict of Interest Statement

The authors declare that the research was conducted in the absence of any commercial or financial relationships that could be construed as a potential conflict of interest.

## Footnotes

1 http://genome.jgi.doe.gov/programs/fungi/index.jsf

2 frodo.wi.mit.edu/

3 http://www.sciencebuddies.org/science-fair-ojects/project_ideas/Genom_GC_Calculator.shtml

## Supplementary Material

**TABLE S1 | Primers to amplify motifs in *T. atroviride.***

**TABLE S2 | Primers to amplify motifs in *T. harzianum.***

**TABLE S3 | Primers to amplify motifs in *T. ressei.***

**TABLE S4 | Primers to amplify motifs in *T. virens.***

**TABLE S5 | Accession number, source of isolation and place of collection of different *Trichoderma* isolates used in the present study.**

**TABLE S6 | A list of unique motifs obtained in the transcript sequences of four *Trichoderma* species analyzed.**

**TABLE S7 | Table showing amino acid runs and number of loci amplified by trinucleotide SSRs.**
