# Supplementary material for: A Comprehensive Characterization of Simple Sequence Repeats in the Sequenced Trichoderma Genomes Provides Valuable Resources for Marker Development
Source: Front Microbiol. 2016 Apr 27;7:575. doi: 10.3389/fmicb.2016.00575 (PMC4846858; doi:10.3389/fmicb.2016.00575)
Supplement: Supplementary file 5 [file Table_5.DOCX]

| **S. No** | **Code** | **Accession number** | **Species** | **Source** | **Place** |
| --- | --- | --- | --- | --- | --- |
| 111 1 | *Th* 01729 | NAIMCC-F-01729 | *T.harzianum* | Soil | Jabalpur |
| 2 | *Th* 01730 | NAIMCC-F-01730 | *T. harzianum* | Rhizosphere of *Cocos nucifera* | Kerala |
| 3 | *Th* 01731 | NAIMCC-F-01731 | *T. harzianum* | Cultivated soil | Maharastra |
| 4 | *Th* 01732 | NAIMCC-F-01732 | *T. harzianum* | Soil | Gujurat |
| 5 | *Th* 01733 | NAIMCC-F-01733 | *T. harzianum* | Waste water & sludge from alkali pulp of rice straw | West Bengal |
| 6 | *Th* 01735 | NAIMCC-F-01735 | *T. harzianum* | Field soil *Lycopersion esculentum* | Pantnagar |
| 7 | *Th* 01738 | NAIMCC-F-01738 | *T. harzianum* | Sugarcane | Coimbatore |
| 8 | *Th* 01741 | NAIMCC-F-01741 | *T. harzianum* | NRC Soybean | MP |
| 9 | *Th* 01742 | NAIMCC-F-01742 | *T. harzianum* | Rhizospheric soil of Pumpkin | Andaman & Nicobar island |
| 10 | *Th* 01745 | NAIMCC-F-01745 | *T. harzianum* | CTCRI Thiruvanantha-puram | Thiruvanantha-puram |
| 11 | *Th* 01746 | NAIMCC-F-01746 | *T. harzianum* | CTCRI Thiruvanantha-puram | Orissa |
| 12 | *Th* 02033 | NAIMCC-F-02033 | *T.harzianum* | Soil | Bharatpur, Rajasthan |
| 13 | *Tv* 01819 | NAIMCC-F-01819 | *T. viride* | Soil of western ghats of Karela | Karela |
| 14 | *Tv* 01820 | NAIMCC-F-01820 | *T. viride* | Soil of western ghats of Karela | Karela |
| 15 | *Tv* 01824 | NAIMCC-F-01824 | *T. viride* | *Triticum aestivum* rhizosphere | New Delhi |
| 16 | *Tv* 01825 | NAIMCC-F-01825 | *T. viride* | Garden Soil | Varanasi |
| 17 | *Tv* 01798 | NAIMCC-F-01798 | *T. viride* | Fibre of *Corchorus* sp. | Calcutta |
| 18 | *Tv* 01800 | NAIMCC-F-01800 | *T. viride* | Wet land soil | Pattambi India |
| 19 | *Tv* 01801 | NAIMCC-F-01801 | *T. viride* | Soil | Vellayami |
| 20 | *Tv* 02047 | NAIMCC-F-02047 | *T. viride* | - | Andhra Pradesh |
| 21 | *Tv* 02048 | NAIMCC-F-02048 | *T.viride* | - | Andhra Pradesh |
| 22 | *Tv* 02502 | NAIMCC-F-02502 | *T.viride* | Black pepper | Andaman and Nicobar Island |
| 23 | *Tv* 02503 | NAIMCC-F-02503 | *T. viride* | Black pepper | Andaman and Nicobar Island |
| 24 | *Tv* 01959 | NAIMCC-F-01959 | *T. viride* | Sukna forest soil | West Bengal |

**Supplementary table 5: Accession number, source of isolation and place of collection of different *Trichoderma* isolates used in the present study:**
