# Supplementary material for: A Comprehensive Characterization of Simple Sequence Repeats in the Sequenced Trichoderma Genomes Provides Valuable Resources for Marker Development
Source: Front Microbiol. 2016 Apr 27;7:575. doi: 10.3389/fmicb.2016.00575 (PMC4846858; doi:10.3389/fmicb.2016.00575)
Supplement: Supplementary file 6 [file Table_6.DOC]

**Supplementary table**

**Supplementary table 6: A list of unique motifs obtained in the transcript sequences of four *Trichoderma* species analyzed**

|  | ***Ta*** | ***Th*** | ***Tr*** | ***Tv*** |
| --- | --- | --- | --- | --- |
| Di |  |  | cg |  |
| Tetra | attt,gcac ,gggt ,  ggtc ,tgcg | aagt ,aatt, acga ,  acgt, actt, agct,  agtc,agtt, attt, ccgt,ctag, gaac, gagt, gata,gcgt, gcta,gtat,gtca, gtcc,gtcg,gtta, tgaa,tgag,ttaa, ttcg | aacg, cgtg, gacg, tact | agga,ataa,cacc, cgat, tcgt,tcta |
| Penta | aaagg,aacga, aagag,aagcg, aattc,actca, agccc,aggcg, atagc, atagg, atcca,atttc, cacgg,cagaa, cagga, catgg, cccaa,cccta, ccgtg,cgact, cgaga, cgctg, ctcgt, ctgaa, gaaac, gacag, gagtt, gatgc, gcagc, gcttt, ggaca, ggcag, ggccg, gggct, ggtac, gtctc, gtggt, gtttg, gtttt, tacag, tatgg, tgctt, ttgcg | aaaac,aaaat, aaacg,aaaga, aaagc, aaatc, aacaa, aactg, aagac, aatag, aatga, aatgt, aattg, acaag, acatc,accaa, acgag, acggc, acgtc, actcg, actct, actgt, agaac, agacg, agatg, agcaa, agcag, agcgc, agctc, agctt, agtcc, agtgc, ataat, ataca, atatg, atccc, atgag, atgcc, attgc, attgt, atttt, caaac, caaag, caaca, caacc, caagc, caatc, caatg | aaggg, acaac, acgaa, caaaa, caccg, catgt, cctca, cgccg, cgcgt, ctgcc, gactc, gcaca, gcagg, gcgaa, ggcgc, ggctg, gtctg, tacct, tagga, tccga, tcgcc, tgggc, tgtat, ttatt | aagga, aataa, aatac, acagt, acgac, acgca, acttc, agatc, agcac, atcag, atgga, atgtc, attcc, catac, cccct, cccga, cgcaa, cgcct, ctact, gacga, gagac, gagag, gaggg, gatca, gatga, gcgag, gcggc, gctgg, ggctc, gggtg, taaca, tgccg, tggat, tgtga, ttcaa, tttat, tttcc, tttgg |
| Hexa | aaaacc, aaacgc, aacaat, aacaca, aacagc, aacgga, aagaat, aagagg, aagcca, aatgaa, acacga, acatca, accggc, acctcc, acggac, acggag, acttcc, agcaca, agcgac, agctcc, agctgg, aggaaa, aggata, aggcac, aggcag, aggcat, gtcgca, agttca, ataccg, atcatg, atcccc, atctac, atgaag, tgacga, tggcaa, atgtcg, caaagc, caacga, caccga, acctgc, agagcc, caggag, caggcg, caggga, catggg, ccaaaa, ccaggg, ccatct, ccatga, ccgact, ccgtac, cctgga, cgacgt, cgagag, cgatgc, cgccaa, cgccag, cggcgc, cggtca, cggtct, cgtcgc, ctactg, tctgac, ctgaaa, ctgagc, ctgcaa, ctggag, ctttcg, gaactg, gaagcg, gaagct, gacacc, gactga, gagacg, gagatg, gagcag, gagggg, gatcat, gatggg, gatggt, gattca, gcacaa, gcagcc, gcagcg, gcatca, gccaca, gccact, gccagc, gcccca, gcgatg, gcggct, gctcac, ggaaag, gaaatg, ggaagg, ggacag, ggcagg, ggcagt, ggccaa, ggcccc, ggcggg, gggaca, gggacc, ggtgat, gtacat, gtaggg, gtcacc, gtgggc, gtgttt, gttggt, tatatg, tatgga, tcactc, tcagaa, tcagat, tccgac, tctacc , tctagc, tctatc, tctcgc, tctgat, tgacaa, tgacat, tgaccg, tgaggc, tgaggg, tgatgg, tgatgt, gcaatt, tgccac, tgccag, tgctgt, tggaac, tggaga, tggcat, tggcga, tggctg, tgggcc, tgtctg, ttcagc, ttcagg, ttccct, ttgcaa, tttgcc | aacaca ,aacagt ,  aaccaa ,aactcg  aagacc , aagagc,  aagcag, aatccc,  aatggg, aattac  acaagg, acactt,  acagcc, acatcc, acatga, acctac, acctgc, acgcag, acgtgt, actctc, actgct,agaaac, agacgc, agccgc, agcgtg, agctcc, aggaac, aggaag,  aggacg, aggaga, aggcca, aggccg, aggctg, agggaa, agggca, agggcg, atcgca, atctca, atgcaa,caacat, caacca, caacct, caaggc, aaggga, caaggt, cacaag, cacgca,cagaca, cagacg, cagctt, caggtc, catcgt, catgac, catgtc, catttg, ccaatt, ccagac, ccagcg, ccagtg, cccacg, cccagt, ccgaca, cgcgac, cgtgac, ccgtgc, cctatg, cctgag, cctttt, cgaaac, gaagac, cgacat, cgagat, cgagcc,gcaact, cgcatt, cgccct, cgccta, cgcctc, cgcctt, cggtgc, ctaagg, ctacat, ctcaca, ctcact, ctcggg, ctctca, ctgaac, ctgcat, ctgcgg, ctgcgt, ctgtac, ctgtcc, ctgtga, cttggg, cttgtt, cttttc, gaaaca, gaaggt, gaagtt, gaatct, gacaat, gaccgc, gacgcc, gagaca, gagact, gagtct, gagtga, gatcct, gatgcc, gatgcg, gatgtc, gcaaag, gccaaa, gccata, gccatt, gccgat, gcgaga, gcgccg, gcggta, gctaca, gcttca, ggaaac, ggacat, ggaccg, ggacct, ggagaa, ggctca, gggaaa, gggcgt, ggtcaa, ggtctg, ggtgaa, gtacag, gtacca, gtacct, gtagcc, gtctca, gtgatg, gtggag, gtgtct, gttcgg, gttgag, taacgg, tcaccc, cacgat, tcatct, tcccac, tcctgt, tcgact, tcgcaa, tcgggg, tcgtct, tctcag, tctgac, tcttca, tcttcc, tgatat, tgatcg, tgcatc, tgccat, tgcgga, tgctac, tgcttt, tggaag, tggcaa, tggcct, tggctc, tgggcg, tggtac, tggtgc, tgtttc, ttcaaa, ttcatc, ttcgtg, ttctcg, ttctga, ttgcga, ttgtcc, tttgac, tttgca, ttttgt | aaagcc, aacctc, aacgac, aacggt, aactgc, aagtcc, aatacc, acaacc, acacgc, acactc, accatc, accatg, acccag, acgaca, acgagg, acggcg, acgggc, actaca, actcga, acttcg, actttt, agaggg, agcaat, agccat, agcgac, agctac, aggcgg, agggca, aggtac, agtcac, atcacg, atttgc, caaccc, caactc, caagga, caaggc, caagtc, cacacg, caccaa, cacgag, cacggc, cacggg, cacggt, agaaac, cagagg, cagtcg, cagtgg, catcag, catcct, catcgg, catcta, ccacgg, ccaggt, cccgct, ccgtgc, ccctcc, ccgaag, ccgacc, cgacgc, ccgagc, ccgcaa, ccggag, ccggca, ccgggt, ccgtcg, cctcat, ctcgtc, cctctc, cctctt, cctggt, cctgtg, ccttcg, cgacca, cgacct, cgagcc, cgaggg, cgatca, cgcacc, cgcagc, gcagtc, cgcatg, cgccat, cgccgt, cgcgac, cgcgat, cggcac, cggccc, cggcct, cggctc, cggctg, cgtctg, cgtgac, ctacaa, ctacag, ctactc, ctcagt, ctcccc, ctctgt, ctgcct, ctgcga, tgtctc, ctgtgc, cttcta, gaacca, gaagga, gaccaa, gaccag, gacccg, gaccct, gacggc, gagaac, gagcac, gattcg, gcaagg, gcaatg, gcaccg, gcacgc, gcagga, gccaat, gccaga, gcccac, gccccg, gccgca, gccgct, gccggt, gcctcg, gcgaag, gcgagg, gcgcca, gcgccc, gcgggc, gcgtct, gctccg, gctcgg, ggacaa,ggacgt, ggaggt, ggatga, ggcacc, ggcgca, ggcgga, gggaag, gggcgc,ggggag, ggtaga, ggtcac, ggtgac, gtcccc, gtcgca, gtgccg, gtgcgg, gtgtcc, tacgga, tcaaca, tcaagg,tcaagt, tcagac, tcatcg, tccaaa, tcccgc, tcctcg, tcgacc, tcgcag, tcgccc, tcgccg, tcggcc, tcgggc, tcggtg, tcgtgg, tctcct, tctgcg, tgaggt, tgatca, tgccgc, tgcctc, tgcggt, tgctag, tgctcc, tggata, tggccg, tgggat, tggggc, tgtgcg, ttcctt | aaacgg, aaagag, aacaaa, aacgag, aagaac, aagctc, aaggcg, aatgca, aattca, aattgg, acaacg, accaag, accagg, acgaag, acgatc, acgtga, actcag, actggc, agacag, agagaa, agagcc, agagtg, agcgag, agcgcc, aggaca, agggca, agtctc, atacac, atctgg, atgcct, atggcg, atggtg, caaaaa, aacctc, cacctc, cactgg, cagaac, catagc, catcca, catccg, catctt, ccaatg, ccagaa, cccaat, cccagc, cccatg, cccggc, ccgaga, ccggga, cctcga, cctctc, cctgat, cctgca, cgaagc, cgaagg, cgacaa, cgacag, cgcccg, cggcat, cggctt, cgggtg, ctcagc, ctcagg, ctcggc, ctgagg, ctggaa, ctggta, cttcgg, gaaaag, gaaaat, gaaagg, gaacgt, gaatca, gacgga, gactca, gagagc, gaggga, gaggtc, gatcgt, gatgga, gcaaga, gcacgg, gcagtc, gcctct, gcgcga,gctacg, gctcag, gctgcg, gctgga, gcttcg, ggagat, ggagcg, ggataa, ggcgaa, ggcgac, gggaat, ggtgtc, gtcaca, gtcgag, gtgtgc, tacaac, tacata, taccac, tattac, tcacca, tccaag, tccgcc, tccgct, tccggc, tcgagc, tctcac, tctctt, tctgaa, tgaaca, tgaaga, tgagag, tgatgc, tgattc, tgcaga, tgcagc, tgccga, tgctct, tgggac, tgaggt, ttttta |
