# Supplementary material for: A Comprehensive Characterization of Simple Sequence Repeats in the Sequenced Trichoderma Genomes Provides Valuable Resources for Marker Development
Source: Front Microbiol. 2016 Apr 27;7:575. doi: 10.3389/fmicb.2016.00575 (PMC4846858; doi:10.3389/fmicb.2016.00575)
Supplement: Supplementary file 7 [file Table_7.DOCX]

**Supplementary Table 7:** Table showing amino acid runs and number of loci amplified by trinucleotide SSRs.

|  | ***Ta*** | ***Th*** | ***Tr*** | ***Tv*** | ***Ta*** | ***Th*** | ***Tr*** | ***Tv*** |
| --- | --- | --- | --- | --- | --- | --- | --- | --- |
|  | **No. of loci** | | | | **Repeat Motif** | | | |
| Ala | 272 | 275 | 518 | 226 | 1185 | 1154 | 2354 | 967 |
| Arg | 342 | 347 | 623 | 311 | 1452 | 1411 | 2741 | 1285 |
| Asn | 33 | 46 | 73 | 28 | 143 | 216 | 336 | 108 |
| Asp | 173 | 176 | 266 | 139 | 715 | 689 | 1173 | 543 |
| Cys | 59 | 57 | 76 | 42 | 243 | 230 | 369 | 174 |
| Gln | 254 | 276 | 528 | 204 | 1211 | 1193 | 2603 | 978 |
| Glu | 213 | 262 | 290 | 238 | 880 | 1059 | 1320 | 1003 |
| Gly | 152 | 128 | 287 | 111 | 609 | 502 | 1258 | 470 |
| His | 87 | 105 | 191 | 60 | 354 | 413 | 848 | 234 |
| Ile | 21 | 34 | 23 | 21 | 75 | 129 | 101 | 79 |
| Leu | 163 | 182 | 270 | 116 | 750 | 737 | 1291 | 469 |
| Lys | 150 | 158 | 140 | 155 | 628 | 668 | 647 | 665 |
| Met | 28 | 23 | 16 | 23 | 112 | 82 | 62 | 92 |
| Phe | 53 | 73 | 31 | 39 | 216 | 272 | 126 | 167 |
| Pro | 164 | 185 | 356 | 160 | 686 | 751 | 1566 | 633 |
| Ser | 232 | 267 | 445 | 179 | 954 | 1096 | 2081 | 728 |
| Thr | 97 | 116 | 234 | 96 | 390 | 494 | 1041 | 379 |
| Trn | 26 | 24 | 29 | 12 | 98 | 91 | 127 | 46 |
| Tyr | 11 | 18 | 11 | 14 | 40 | 69 | 50 | 56 |
| Val | 17 | 46 | 43 | 36 | 65 | 170 | 189 | 139 |
